# A Review of Full-Body Radiography in Nontraumatic Emergency Medicine

**DOI:** 10.1155/2012/108129

**Published:** 2012-12-02

**Authors:** S. P. Whiley, G. Mantokoudis, D. Ott, H. Zimmerman, A. K. Exadaktylos

**Affiliations:** ^1^Department of Human Biology, University of Cape Town, Observatory 7925, South Africa; ^2^Lodox Systems, 7 Dartfield Road, Sandton, Johannesburg 2146, South Africa; ^3^Department of Otorhinolaryngology, Head and Neck Surgery, Insel Hospital, 3010 Bern, Switzerland; ^4^Department of Radiology, Insel Hospital, Freiburgstraße, 3010 Bern, Switzerland; ^5^Department of Emergency Medicine, Insel Hospital, Freiburgstraße, 3010 Bern, Switzerland

## Abstract

This paper reports on the application of full-body radiography to nontraumatic emergency situations. The Lodox Statscan is an X-ray machine capable of imaging the entire body in 13 seconds using linear slit scanning radiography (LSSR). Nontraumatic emergency applications in ventriculoperitoneal (VP) shunt visualisation, emergency room arteriography (ERA), detection of foreign bodies, and paediatric emergency imaging are presented. Reports show that the fast, full-body, and low-dose scanning capabilities of the Lodox system make it well suited to these applications, with the same or better image quality, faster processing times, and lower dose to patients. In particular, the large format scans allowing visualisation of a greater area of anatomy make it well suited to VP shunt monitoring, ERA, and the detection of foreign bodies. Whilst more studies are required, it can be concluded that the Lodox Statscan has the potential for widespread use in these and other nontraumatic emergency radiology applications.

## 1. Introduction

The Lodox Statscan machine is a device capable of obtaining full-body radiograms using a unique, linear slit scanning radiography (LSSR) technology. Since first being introduced to the medical community more than ten years ago [1], it is now installed in nearly 50 sites worldwide.

The Statscan was originally developed from a technology used to screen mine workers in South Africa for smuggled diamonds. It is intended for use in trauma and emergency wards as a replacement for the traditional basic Advanced Trauma Life Support (ATLS) set of plain radiographs called for in the Primary Survey [2, 3]. Its use in polytrauma screening in cases of motor vehicle accidents, falls or violent trauma has been documented by several groups [2, 4–8]. However, during its years of use, innovative clinicians have uncovered more varied uses for the technology that have proven useful in nontraumatic emergency medicine. 

## 2. Methods

### 2.1. Search Strategy and Literature Selection

We searched MEDLINE for English language articles (1966–2012) using terms for LODOX, Statscan, or full-body radiography. There was no search restriction for age or publication date. A full-text screening was performed for all papers found by our search strategy. We excluded nonenglish language papers and articles with no data about full-body radiography with no relation to emergency medicine, or with a full-body radiography application only to trauma patients.

### 2.2. System Overview

The Lodox Statscan consists of an X-ray tube and charge-coupled device (CCD) detector, mounted on opposite ends of a C-arm. The C-arm is attached to the system base unit, which is anchored to the floor. The patient is placed on a gurney which is “docked” to the base unit before scanning. During scanning, the C-arm moves from the head to the foot of the patient at up to 138 mm/s, emitting a thin, pencil beam of X-rays which are detected by the narrow detector. This configuration (known as LSSR) eliminates most of the scatter associated with conventional fan-beam radiography and accounts for the very low radiation dose required for the machine to form X-ray images. The C-arm can also be rotated axially from AP (0°) to Lateral (90°), or any oblique position in between, and the table can accommodate Trendelenburg tilting of 10° on either side, to allow images in different planes to be obtained without movement of the patient [4].

The low radiation dose of the machine has been examined in previous studies, and it has been found that a full-body radiograph emits less than 75% of the dose that a conventional chest X-ray emits [4, 9]. In comparison to the usual trauma panel of AP chest, AP pelvis and Lateral cervical spine, a full-body AP radiograph and lateral spine radiograph on the Lodox unit exposes the patient to less than 10% of the radiation dose [9]. In a study measuring entrance dose and effective dose (E) using the Lodox Statscan, Irving et al. found that they were reduced by 70% and 85%, respectively, in comparison to conventional radiography, as reported by the United Nations Scientific Committee on the Effects of Atomic Radiation (UNSCEAR) [10].

It has also been shown that the time required to screen a patient is less using the Lodox Statscan (full-body scan) than when using conventional radiography (routine ATLS X-rays) by an average of 14.1 minutes (9.8–22) [5, 8, 11]. However, both Exadaktylos et al. [8] and Boffard et al. [5] find that total reduction in resuscitation time when using the Statscan was negligible, although neither discusses possible reasons for this.

The Lodox Statscan images have been shown to have the same, or better, image quality as conventional X-ray, despite the speed and low radiation of imaging [4, 5, 7, 11], with Deyle et al. [7] finding a sensitivity of 62% and specificity of 99%.

These unique features of the Statscan (full-body image, low dose, fast time, high image quality) have indicated its use as the primary scanning mechanism in trauma imaging. However, they have also provided the basis for new applications of the technology in nontraumatic trauma.

## 3. Results

Our search strategy identified 30 citations. Seven papers were excluded after full-text search according to the predefined criteria. 14 papers contained background and technical information relevant to the Lodox Statscan, but not to nontrauma applications. Nine manuscripts were selected as containing information regarding nontrauma radiography with Lodox (see Table 1). Within these, four different radiographical applications were identified.

### 3.1. Ventriculoperitoneal Shunts

Ventriculoperitoneal (VP) shunts are commonly used to treat hydrocephalus in adults and children. Despite improvements in the use of VP shunts, there is still a very high rate of complications [12, 13]. Patients are prone to a varied range of malfunctions including obstruction, rupture, disconnection at a junction, or migration of the catheter [14, 15]. Shunt malfunction usually presents with acute neurological and other symptoms, and as such, patients usually present to emergency wards. To identify VP shunt malfunctions, extensive radiographic imaging is required, generally consisting of a series of overlapping plain radiographs that trace the trajectory of the catheter from the head to its end, in order to check for positioning and patency along its length. Conventionally this involves radiographs of head, neck, chest, and abdomen, with adjacent areas overlapping to ensure complete visualisation. Furthermore, patients are also often subjected to multiple Computed Tomography (CT) examinations. This imaging, often repeated many times over a patient's life, contributes to an increased exposure to ionising radiation and the associated risks of cancer [16–18].

**Figure 1:**
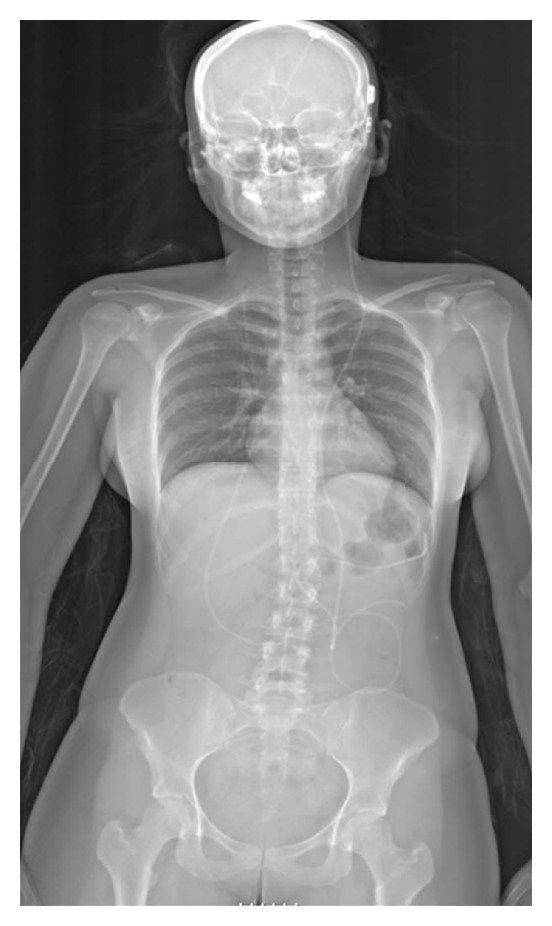
Lodox radiograph showing a ventriculoperitoneal shunt.

The research group at Inselspital, Bern have reported a new technique of VP shunt visualisation using the Lodox full-body scanner [19, 20]. The ability of the scanner to obtain images of the entire patient in one scan eliminates the positioning difficulty of conventional radiography, and the increased radiation incurred when imaging overlapping areas. Combined with the scanner's inherently low dose, this has allowed fast, accurate imaging of the shunt, with much reduced radiation.  Figure 1 shows a full-body Lodox scan, allowing the path of a VP shunt to be traced in its entirety. Fathi et al. found a statistically significant difference in time and number of scans using conventional X-rays versus Lodox with scan time, on average, 12.58 minutes faster with the Lodox (8.8 min versus 21.28 min) and 3.9 more images taken with conventional imaging (2.4 versus 6.3) [19]. The Lodox images provided the same amount of clinical information as the conventional series. The authors conclude that it is a valuable technique for reducing dose to the patient and time spent in discomfort during imaging. Pitcher et al. also report that the Lodox technique of imaging VP shunts is routinely used at their institution [21].

### 3.2. Arteriography

Another application of the Lodox Statscan in nontraumatic emergency radiology is in the context of emergency room arteriography (ERA). ERA consists of a single injection of intra-arterial contrast proximal to the area of interest, followed by X-ray imaging [28]. Its advantages are that it is simple to perform and can be (and usually is) performed by staff in the emergency centre, without the need for specialist imaging equipment or staff, or the transport of patients [29]. ERA allows patients with suspected peripheral arterial injuries to be evaluated in the emergency room before further procedures or potential premature discharge [28]. 

**Figure 2:**
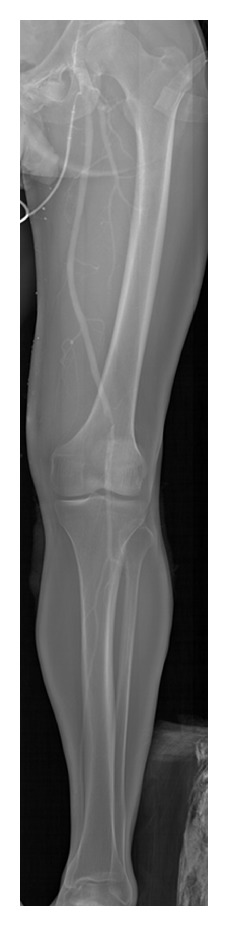
Example of Emergency Room Arteriography using the Lodox Statscan. Notice the occlusions at the proximal region of interest, and distal to this.

ERA was first introduced in 1958 [30] and since then has undergone few modifications. However, a group in Groote Schuur hospital, Cape Town have applied the full-body imaging capabilities of the Lodox Statscan to the technique [26]. In a two-year retrospective study on patients undergoing ERA, they found that the sensitivity and specificity of Lodox ERA were both 100%. The Statscan ERA cleared 30% of patients from further investigation by showing no pathology. Furthermore, it directly altered patient care in 20% of cases, due to the fact that pathology both in, and distal to, the site of interest was imaged in a single scan, thus diagnosing unsuspected distal arterial emboli.  Figure 2 shows ERA of a lower limb performed using the Lodox Statscan. The authors find the Statscan a safe, rapid, simple, and accurate tool for performing ERA, and it is now the standard protocol for ERA in their institution.

### 3.3. Foreign Bodies

Ingestion of foreign bodies, and the associated complications, is a common presentation in emergency wards [31]. Patients are most commonly children, with adults largely presenting in cases of mental handicaps, psychiatric disorders or substance abuse and smuggling [23]. Complications can be severe, with possible injuries to airways, oesophagus, or gastrointestinal (GI) organs. Precise management is critical, with a high number of cases ending in mortality [23, 31–33]. Indications for investigation are plain radiography (or metal detection in the case of metallic objects), followed by endoscopy if required, and CT imaging when indicated [34, 35].

**Figure 3:**
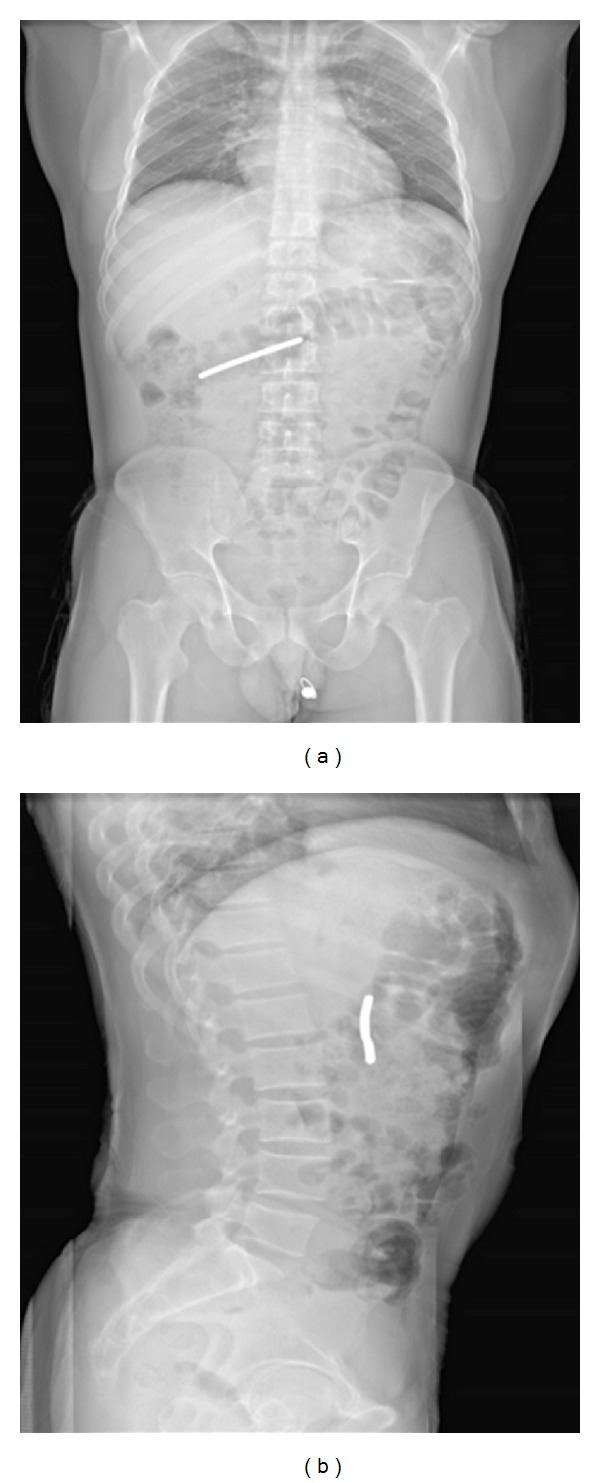
Lodox radiograph showing (a) the AP and (b) the lateral view of an ingested foreign object.

Mantokoudis et al. [23] report on the use of full-body X-ray scanning in the detection of foreign bodies. In a retrospective comparison between chest X-rays and Lodox scans, sensitivity, specificity, and irradiation were evaluated. They found the Lodox scans to have greater accuracy in detecting foreign bodies than the chest X-rays (sensitivity 90%, specificity 100% with Lodox versus 44.4% and 94.1%, resp., with chest X-rays) and suggest that this is due to the greater area of visualisation using the Lodox scans.  Figure 3 shows AP and lateral Lodox images of an ingested foreign body that would not have been visible in a conventional chest X-ray view.

In a case report from the same institution, the specialised situation of detecting drug smuggling in “body packers” was presented [22]. In this case, a suspected drug smuggler, arriving at the emergency ward after a fall, was imaged using the Lodox scanner, revealing five packages in the cecum and ascending colon, prompting further imaging. The authors recommend the use of full-body imaging for this unique ER purpose, both to expedite diagnosis, and reduce radiation to the patient.

### 3.4. Paediatric Imaging

Several authors have reported on the use of the Lodox system for imaging in paediatric emergency wards [21, 24, 27, 36–38]. In most of these situations, the system was used for traumatic emergencies, largely due to its dose-saving quality, and the particular benefit this has in paediatric cases [39], although Evangelopoulos et al. [37] did not find this dose benefit in their study of 19 paediatric patients, possibly due to the inclusion of lateral full-body scans.

**Figure 4:**
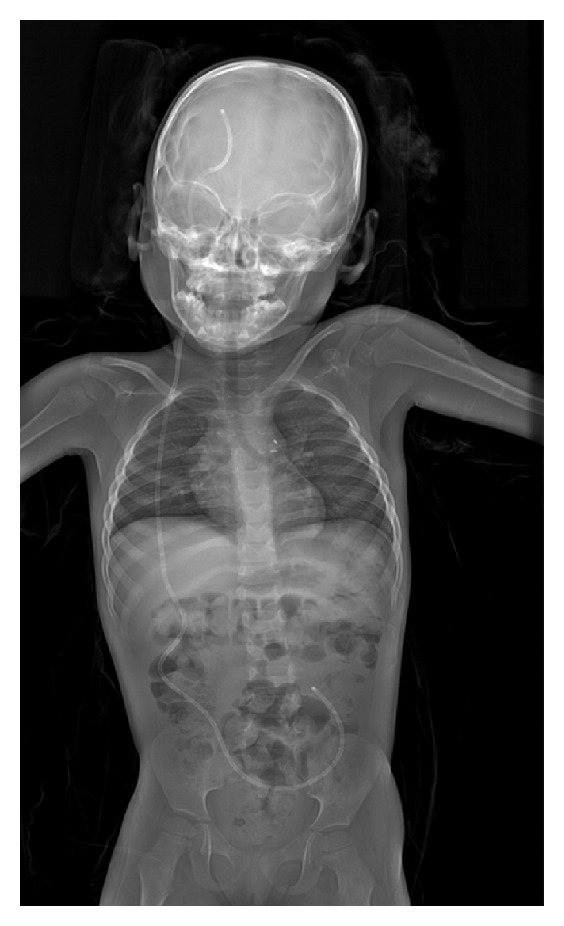
Example of the use of Lodox in VP shunt visualisation in paediatric patients.

However, apart from paediatric trauma imaging, the Statscan has also been used in nontrauma situations. The use of Lodox in imaging VP shunts has been discussed above. VP shunt malfunction is a condition frequently found in children, and Figure 4 shows a Lodox radiograph of a paediatric VP shunt patient.

**Figure 5:**
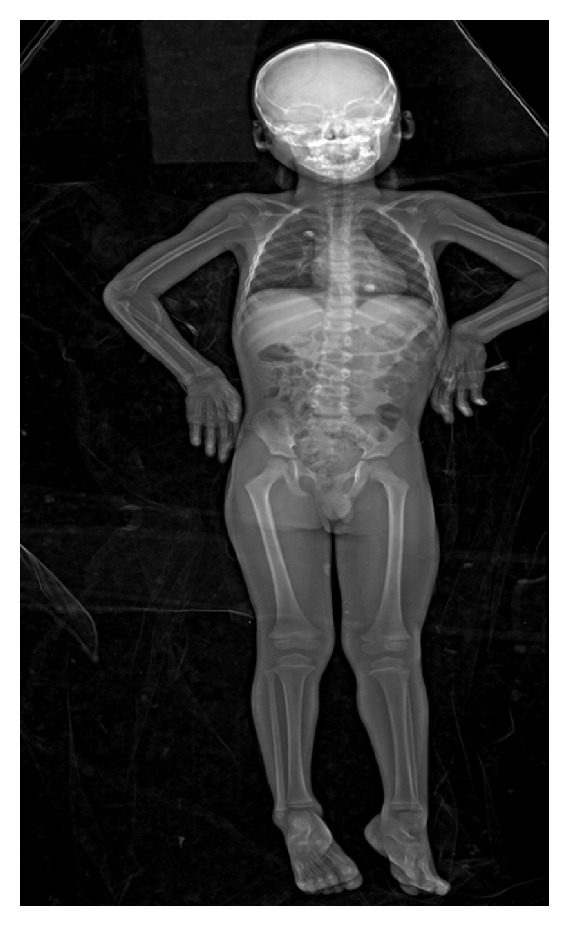
Paediatric full-body X-ray image showing superior imaging of the major airways.

In their pilot study on the use of the Lodox Statscan at the Red Cross Children's Hospital, Pitcher et al. note the modality's superior ability to visualise the three major airways in children [21]. A further study on erect chest radiography in the same institution on 33 children confirmed this and advocated the use of the Statscan in the diagnosis of childhood tuberculosis (TB), where airway narrowing occurs as a result of nodal compression [24].  Figure 5 shows a Lodox supine radiograph, illustrating the superior ability to visualise the major airways. Diagnosis of paediatric TB is difficult and can be a large and burdensome part of paediatric trauma, especially in sub-Saharan Africa where prevalence of the disease is high [40]. The potential of the Lodox to improve imaging in this area of nontrauma emergency could be invaluable [21].

As well as paediatric chest imaging, the Lodox Statscan has been used to acquire full-body X-rays of paediatric patients. Its lower dose and fast imaging time (requiring less time for the patient to be still) is uniquely suited to paediatric orthopaedic studies. The Lodox system has been found to be useful in the assessment of nonaccidental injuries, bone dysplasia, and leg length discrepancies [41]. The Lodox viewing software, which allows distortion correction and accurate geometric measurement, has been shown to have higher interobserver reliability than previously recorded, which gives it the potential to assess and document a variety of nontraumatic paediatric pathologies [21, 27].

## 4. Discussion

Nontraumatic emergencies form a core and important group of patients in all emergency departments. As with traumatic emergencies, treatment of these patients is critical, and time is usually of the essence. Without accurate information on the condition of the patient, physicians are unable to respond appropriately, and medical imaging is most often the tool used to provide this information. The Lodox Statscan full-body X-ray scanner has been designed and developed specifically to provide this imaging information in emergency centres [1], and it has been reported as effective in doing so in terms of both speed of imaging, and quality of images [5]. Furthermore, through the use of LSSR technology, the scanner provides these images at a very low radiation dose [9]. 

In this paper we have discussed the specific use of the Lodox system in nontraumatic emergencies. In ventriculoperitoneal shunt patients, the ability of the scanner to provide an image of the entirety of the shunt has been shown to be beneficial in diagnosing malfunctions throughout its length [19]. This same ability to scan the full-body allows emergency room arteriography to be performed to a much higher degree of effectiveness than previously, due to the Lodox images showing the full arterial structure, but requiring only a single image and a single injection of contrast [26]. Considering the dose saving to both patient and practitioner, it seems possible that this type of technology could also be useful in conventional arteriography examinations, although this has not been studied.

In the detection of foreign bodies, it is again the large format images that provide the benefit, allowing ingested objects situated outside the conventional field-of-view of a chest radiograph to be imaged in the first instance, thus eliminating the need for further imaging to localise the objects [23]. In addition, investigations using Lodox reduce the radiation dose received per patient by a mean of 65% (184 *μ*S with Lodox versus 524 *μ*S with chest X-rays [23]). Part of this lower dose contribution is due to the Lodox LSSR technique. However, a further, substantial dose reduction is largely due to the fact that when conventional chest radiography is used as the primary method of evaluation, additional X-ray images are often required to visualise, or rule out the presence of, the foreign body, whereas these are not necessary when a full-body radiograph has been obtained. Even in the case of drug smugglers, where ingestion and the associated symptoms have been deliberate, this type of technology is beneficial in accelerating diagnosis (and perhaps even conviction). However, radiography should best be avoided in cases with a clear patient history and persistent symptoms after ingestion of a foreign body since the gold standard is endoscopy [34].

Paediatric imaging on the Lodox Statscan has been more extensively studied [21, 24, 27, 36–38] and the benefits of speed and low dose in the context of children seem well accepted. Extension of the imaging from traumatic emergencies to chest imaging (particularly for early paediatric TB diagnosis), and skeletal studies has been suggested [21]. However, again it seems that just a few centres are pioneering these techniques, and comprehensive studies and trials proving their efficacy are yet to be performed, despite the clear indication of their benefit in paediatric emergency medicine.

Some other potential uses for the Lodox in nontraumatic emergencies have also been suggested in the literature, such as the imaging of urinary stones [25] and the imaging of pregnant women [42]. Whilst a pilot study on phantoms has been performed on the former, and anecdotal evidence exists about the possibilities in the latter, there remains scope for some large-scale studies and trials to objectively illustrate these benefits.

### 4.1. Limitations

Despite the benefits illustrated for the use of Lodox full-body imaging in all of the reported cases and despite the broad spectrum of indications for a low-dose full-body scan, there are fewer than 50 systems installed internationally, and only a few of these centres have conducted studies and reported on these novel imaging techniques. More extensive trials are needed to show the efficacy of these full-body X-ray techniques in nontraumatic emergencies. Image quality is comparable or even superior to conventional X-ray images. However, pulsation artefacts of the heart occur since the c-arm moves along the patient during image acquisition and captures the heart at various phases [23] (although no blurring occurs during this process and radiologists can become accustomed to reading whole body radiographs). A further limitation is that the Lodox Statscan is a nonportable device and cannot be applied at the bedside in intensive care units or in patient rooms in the Emergency Department. In addition, patients are forced to remain in a lying position. Although this can be an advantage for the elderly or for children, nonintubated patients with breathing disorders or with a tracheostoma might face difficulties.

### 4.2. Indications and Contraindications for Full-Body Radiographs

Evangelopoulos et al. [2] published a protocol for the use of Lodox full-body radiography within an emergency trauma setting, which included using ultrasound together with the full-body X-ray image to provide initial information on soft tissue, as well as bony, pathologies as adjuncts to the primary ATLS survey. However, to the authors' knowledge, no previous publications have discussed a protocol for the use of the Lodox Statscan in nontrauma emergency medicine. What is clear from the studies reported on in this review is that full-body radiographs can be useful for imaging of ventriculoperitoneal shunts, Emergency Room Arteriography, detection of foreign bodies and several paediatric applications. While the ionising radiation that the patient is subjected to with the Lodox is much less than that of conventional imaging [9], it is still true that no radiation is preferable to even minimal radiation, so that if clinical judgement indicates no need for medical X-ray imaging, full-body radiographs should be avoided. In cases where a single, focused radiograph (e.g., a chest X-ray) is considered sufficient for diagnosis, the scanning window of the Lodox should be limited to contain only the region of interest. There is no need to scan the entire body and therefore expose patients to more radiation than necessary.

Furthermore, as with all plain X-ray techniques, there are limits to the specificity and sensitivity of 2D images, and further imaging (whether involving ionising radiation or not) may be indicated in complicated cases. For instance, in arteriography, Lodox is not suggested as a replacement to the angiography suite, but as a preliminary step that can be performed at speed in an emergency situation in order to better prepare clinicians for treatment of the patient. However, Lodox imaging has never been used for coronary or cerebral angiography, and would be unlikely to provide diagnostic images in these cases. Likewise, during evaluation of the patency of a VP shunt, further imaging such as CT, MRI or sonography might be called for in order to properly treat the patient [13]. A Lodox full-body radiograph might be useful in indicating the need for these, and location for imaging, but cannot replace them. Indeed, a head CT is often one of the preferred protocols when evaluating VP shunt complications in the cranial region and would need to be performed in addition to the full-body radiograph, as it would if a series of plain X-ray images had been used. In terms of paediatric imaging, any dose-saving is highly valued, so in cases where the need for plain X-ray imaging was not indicated, or would be a superfluous addition to other imaging studies, then the acquisition of a full-body radiograph would not be recommended.

Since the Lodox Statscan scanner is the only full-body X-ray machine currently produced or sold, it is difficult to compare the studies reported here with similar, relevant studies using conventional imaging. What can be seen is that the unique ability it provides to obtain full-body radiographs in one sweep, at high speed, and with a low-dose penalty to patient or practitioner could provide a unique tool for treating patients, although results from a greater number of emergency centres need to be published.

## 5. Conclusion

The Lodox Statscan full-body X-ray scanner has been shown to have benefits in imaging of a variety of nontraumatic emergency situations. While initial trials and studies show the advantages of the novel use of this technology, it remains to be seen whether more centres will adopt these new techniques or pioneer further innovations in the use of full-body scanning.

## Figures and Tables

**Table 1:** Publications of full-body radiography applications to nontraumatic patients.

1st Author/year	Baseline population	Study method	Number of patients	Full-body radiography application
Klenke, 2012 [22]		Case report	1	Foreign body detection
Mantokoudis, in press [23]	Adults with ingested foreign bodies	Cross-sectional	38	Foreign body detection
Fathi, 2011 [19]	Adults with suspected VP shunt malfunction	Comparative study	46	Ventriculoperitoneal shunts
Daya, 2009 [24]	Pediatric patients	3-month comparative study	33	Paediatric erect chest imaging
Szucs-Farkas, 2009 [25]		Phantom study		Urinary stones
Pitcher, 2009 [21]	Pediatric patients	3-year review	867	Pediatric imaging, major airways, childhood tuberculosis
Ball, 2007 [26]	Adults with suspected vascular occlusions	24-month retrospective review	10	Arteriography
Schaller, 2007 [20]	Adults with suspected VP shunt malfunction	Report on trial series	8	Ventriculoperitoneal shunts
Douglas, 2007 [27]	Paediatric patients	Comparative study	101	Paediatric imaging, orthopaedic measurements
